# The Worries of Out-of-Home Activities in Patients With Inflammatory Bowel Disease: A Survey Study During the COVID-19 Pandemic

**DOI:** 10.1155/2024/6634377

**Published:** 2024-07-03

**Authors:** Yun-Hui Fei, Meng-Hui Zhang, Min-Na Zhang, Zhao Shen, Hong-Gang Wang

**Affiliations:** Department of Gastroenterology The Affiliated Huaian No.1 People's Hospital of Nanjing Medical University, Huai'an, Jiangsu Province, China

**Keywords:** COVID-19, inflammatory bowel disease, out-of-home activity, survey, travel worries

## Abstract

**Background:** Patients with inflammatory bowel disease (IBD) often experience worries related to travel due to frequent bowel movements. However, there is currently limited research focusing on the travel worries of patients with IBD. The aim of this study was to assess the level of worry regarding out-of-home activities in patients with IBD and identify factors associated with worry.

**Methods:** This study included patients with IBD who visited the outpatient clinics between September 2020 and March 2022, during the COVID-19 pandemic. Participants completed a self-designed questionnaire, providing general clinical data and indicating their level of worry for out-of-home activities.

**Results:** A total of 529 patients with IBD completed the questionnaire. Patients with Crohn's disease (CD) had a higher proportion of individuals under 40 years old and males compared to patients with ulcerative colitis (UC). Regarding out-of-home activities, patients with UC expressed greater worry about going out and taking buses than patients with CD. However, there were no significant differences observed between the two groups in terms of travel worries and worries about finding public washrooms. A significant majority (85.4%) of patients with clinically active IBD expressed worries about not finding public washrooms when going out, while 46.7% of patients in clinical remission had similar worries. Moreover, the worry about finding public washrooms was higher in patients with UC compared to those with CD, both during the clinical activity and remission.

**Conclusion:** This survey conducted during the COVID-19 pandemic reported worries among patients with IBD about out-of-home activities. The patients with clinically active IBD, especially UC, expressed worries about not finding public washrooms when going out. We highlight the actual psychological and quality of life challenges faced by patients with IBD.

## 1. Introduction

Patients suffering from inflammatory bowel disease (IBD) often experience chronic gastrointestinal symptoms, including varying degrees of urgency, abdominal pain, diarrhea, joint pain, oral ulcers, eczema, and perianal inflammation [1]. Moreover, many patients also encounter nonspecific symptoms such as fatigue, which can impede their ability to travel and engage in social activities. The COVID-19 pandemic has further exacerbated these challenges, severely affecting the health and daily lives of individuals, particularly those with IBD [2]. Over the past few decades, the incidence of IBD has steadily risen, necessitating concerted efforts from all stakeholders to prevent and manage the disease and alleviate the global burden [3].

Although there is considerable attention given to the ability of patients with IBD to travel, there is a dearth of information regarding the effects of engaging in out-of-home activities. To address this gap, we conducted a questionnaire survey among patients with IBD to gain insights into their current out-of-home activities and worries regarding related events. By providing patients with IBD with practical advice for out-of-home activities and implementing suitable intervention measures, we aim to improve their overall quality of life.

## 2. Materials and Methods

### 2.1. Participants

This study included patients with IBD who sought treatment at the outpatient department of The Affiliated Huaian No.1 People's Hospital of Nanjing Medical University between September 2020 and March 2022. An anonymously designed questionnaire, detailed in the supporting table (available here), was administered to patients aged 14 years or older who have been diagnosed with IBD, to assess their worries related to out-of-home activities.

### 2.2. Study Design

This study was a cross-sectional survey, and data were collected through an online questionnaire. The questionnaire collected general information about the patients, including (1) demographic data—age, gender, and type of IBD—and (2) worries about out-of-home activities, including worries about going out, traveling, taking buses, and finding public washrooms. These questions were scored using a 5-point Likert scale, ranging from “not worried” to “extremely worried.” (3) Worries about not finding public washrooms during clinical activity and remission were answered using a binary-choice method. The English version of the questionnaire can be found in the supporting table (available here). The overall alpha coefficient of the questionnaire was 0.878, indicating good reliability, and the KMO coefficient was 0.876, with *p* < 0.05, indicating good validity. Participants who scored their level of worries at three points or higher on the 5-point Likert scale were classified as “worried.”

### 2.3. Questionnaire

The survey questionnaire was produced in Chinese format. All participants were informed by the relevant medical staff (through unified training) and provided a face-to-face interview to complete the questionnaires. All the questions are mainly self-filled. The quality of the surveys was guaranteed by the on-site quality control method. The main items involved in the questionnaire include the patient's gender, age, disease subtype (UC or CD), disease severity assessment scales, and worry level scores (each item was one to five points that one represents not worried and five represents the highest level of worry), including worried about going out, travel, taking the bus, and finding public washrooms. The disease severity assessment scales included Partial Mayo Score (PMS) and Harvey Bradshaw Index (HBI) [4]. PMS was composed of three categories (bleeding, stool frequency, and physician assessment) rated from zero to three, which were summed to give a total score ranging from zero to nine. Patients with UC were divided into clinical active disease (PMS score > = 3) and clinical remissive disease (PMS score of zero to two). HBI consists of five clinical parameters: general well-being, abdominal pain, number of liquid stools per day, abdominal mass, and complications. The total score was the sum of individual parameters. Patients with CD were divided into clinical active disease (HBI > = 5) and clinical remissive disease (HBI score of zero to four).

### 2.4. Ethical Statement

The Ethics Committee of The Affiliated Huaian No. 1 People's Hospital of Nanjing Medical University approved this study (No. KY-2020-004-02), and informed consent was obtained from all participants.

### 2.5. Statistical Analysis

All data were analyzed using SPSS software (version 26.0; SPSS Inc., Chicago, Illinois). Group comparisons were performed using chi-square tests and *t*-tests. The interactions between the worries and age and gender were evaluated with Spearman's correlation analysis. *p* < 0.05 was considered statistically significant.

## 3. Results

### 3.1. Participant Characteristics

The study included patients with IBD who visited the hospital and were capable of independently completing the questionnaire pertaining to out-of-home activities. Incomplete questionnaires were considered invalid, and the data were not included in the analysis. A total of 529 complete questionnaires were collected. The median age of these participants was 37 years (interquartile range 29–46 years). Of these, 27 patients were under the age of 18, and they completed the questionnaire with the assistance of their guardians. Among the participants, 45.9% were diagnosed with UC, while 54.1% were diagnosed with CD, consisting of 312 males (59.0%) and 217 females (41.0%) (Table 1). Out of these participants, 121 cases of UC and 148 cases of CD were in clinical active disease according to PMS or HBI scores.

### 3.2. Age and Sex Comparison in Patients With IBD

Significantly different age and sex distributions were observed among patients with different types of IBD. The proportion of patients aged ≤ 40 years was higher among patients with CD (73.4%) compared to UC (50.6%) (*p* < 0.001). Additionally, the proportion of males was higher among patients with CD (64.0%) compared to UC (53.1%) (*p* = 0.011).

### 3.3. Comparison of Worry Levels About Out-of-Home Activities in Patients With IBD With Different Characteristics

Patients with UC had a higher level of worry regarding going out and using public transportation than patients with CD (*p* < 0.05). However, there was no difference in the level of worry about travel and finding public washrooms between the two groups (*p* > 0.05) (Table 2).

### 3.4. Correlation Analysis Between Worry Levels and Age and Gender

Was the worry of IBD about out-of-home activities influenced by age and gender? We conducted additional analysis and determined that there was no significant correlation between their worries and age or gender (*p* > 0.05) (Table 3). Our findings suggest that the worries of patients with IBD regarding out-of-home activities primarily stem from the disease itself.

### 3.5. Comparison of Worry Levels About Not Finding Public Washrooms During Different Disease States in Patients With IBD

Participants who scored their level of worries at three points or higher were classified as “worried about not finding public rooms.” A significant majority (85.4%) of patients with clinically active IBD expressed worries about not finding public washrooms when going out. However, a considerable proportion (46.7%) of patients with IBD in clinical remissive disease continued to worry about the accessibility of public washrooms while being out and about. Patients with UC expressed a greater level of worries about the availability of public washrooms while venturing outside, irrespective of their disease states, in contrast to patients with CD (*p* < 0.05) (Table 4).

## 4. Discussion

Our study, involving 529 patients with IBD during the COVID-19 pandemic, revealed noteworthy insights into the psychological impact on patients. Among clinically active patients with IBD, 85.4% expressed concerns about the availability of public washrooms during outings. A distinct pattern emerged, with patients with UC exhibiting higher apprehension about going out and using public transportation compared to those with CD. This prompted reflection on the specific influence of the pandemic on the psychological well-being of patients with IBD.

Patients with UC demonstrated heightened worries related to out-of-home activities, potentially linked to disease activity and characteristics. Due to the prevalence of rectal irritation symptoms in patients with UC, emergency bowel movements may be more common, leading to worries about unforeseeable situations such as the unavailability of public restrooms. This cautious attitude differed significantly from the apparent lack of worry observed in patients with CD, emphasizing the impact of symptoms on daily life. The lower immune defenses in patients with IBD [5], coupled with the pandemic context, led to recommendations for reduced out-of-home activities [6]. While some studies suggested increased gastrointestinal symptoms in COVID-19-diagnosed IBD, the overall risk did not seem elevated [7–9]. Nonetheless, the prevalence of infectious diseases had psychological and economic repercussions for IBD [10]. Considering vaccination status, patients with UC not yet vaccinated exhibited a higher psychological burden, fearing infection during outings. The pandemic altered activity patterns globally, with reduced physical activity and increased concerns about using public transportation [11]. Patients with IBD, particularly those with clinically active disease, faced challenges in finding restrooms outside, significantly impacting their quality of life [12, 13].

Promoting physical activity and public transportation, with due consideration for associated risks, could benefit overall health [14–18]. However, potential risks of gastrointestinal infections during outdoor activities should be acknowledged, especially for patients with IBD. Hypoxia-induced inflammatory mechanisms during air travel posed additional challenges, particularly for patients with UC experiencing urgency in defecation [19, 20]. Medical advice and information dissemination played pivotal roles in shaping worries [21, 22]. Patients with UC receiving detailed information on infection risks during the pandemic were more prone to heightened levels of worries. Fears of contracting the novel coronavirus during restroom use, though plausible, needed careful consideration in light of limited evidence of fecal transmission.

Beyond the pandemic, the worry of finding public restrooms is a longstanding issue for patients with IBD [23, 24]. Various organizations have developed comprehensive maps of public restrooms to assist patients in planning activities. Future research could explore coping strategies during nonpandemic times and assess the practical utility of these restroom maps, providing a comprehensive understanding of patients' needs.

Limitations of our study include potential recall bias and lack of detailed investigation into comorbidities. Mental health plays a role in patient's worried levels. These participants did not report a history of previous diagnosis of anxiety or depression in the questionnaire. Unfortunately, we did not conduct a scale evaluation of the patient's mental health in this questionnaire. There are other aspects to take into consideration when evaluating whether the COVID-19 pandemic played a role in the well-being of patients with IBD. Unfortunately, we failed to compare the level of concern before and after COVID-19 in the same cohort. Another limitation was that the information about COVID-19 vaccination among patients with IBD was not included in this study. This might affect patients' worries about out-of-home activities. Furthermore, the anxiety and depression status of the participants may influence the study's outcomes. While the participants indicated in the survey whether they have been diagnosed with anxiety or depression, the majority of these diagnoses were not made by a specialized hospital. Consequently, relying solely on self-reported data regarding the presence of anxiety or depression may not provide sufficiently accurate information. Future research should address these limitations and consider tailoring outing plans during the active phase of IBD. For patients familiar with online activities, transitioning to online exercises during disease activity or outbreaks of infectious diseases may help maintain their health. Optimizing healthcare management for patients with IBD in their daily lives is crucial to mitigate the negative impacts of travel restrictions during the COVID-19 pandemic and enhance patient adherence.

## 5. Conclusions

This survey conducted during the COVID-19 pandemic reported worries among patients with IBD about out-of-home activities. The patients with clinically active IBD, especially UC, expressed worries about not finding public washrooms when going out. We highlight the actual psychological and quality of life challenges faced by patients with IBD.

## Figures and Tables

**Table 1:** General characteristics of patients with IBD.

**Item**		**Cases (** **n** **)**	**Percentage**
Age, years	≤ 40	333	62.9%
	> 40	196	37.1%
Gender	Male	312	59.0%
	Female	217	41.0%
IBD	UC	243	45.9%
	CD	286	54.1%

*Note:* The data in the table represents the number of patients and the percentage in the total number of patients with IBD.

Abbreviations: CD, Crohn's disease; IBD, inflammatory bowel disease; UC, ulcerative colitis.

**Table 2:** Comparison of worry levels of out-of-home activities in patients with IBD.

**Item**	**IBD**	**UC**	**CD**	**T** ** value**	**p** ** value**
Worried about going out	2.905 ± 1.389	3.050 ± 1.399	2.759 ± 1.367	−2.101	0.036
Worried about travel	3.070 ± 1.439	3.126 ± 1.490	3.015 ± 1.387	−0.766	0.444
Worried about taking the bus	3.103 ± 1.488	3.276 ± 1.511	2.930 ± 1.448	−2.338	0.020
Worried about not finding public washrooms	3.249 ± 1.450	3.342 ± 1.465	3.156 ± 1.432	−1.280	0.201

*Note:* Data presented as mean ± SD. The items of worries included worries about going out, traveling, taking the bus, and finding public washrooms. Each item was quantitatively rated from one to five. It was interpreted as one representing no worries, and five representing the highest level of worries. This table presents a statistical comparison of the worry levels of out-of-home activities between UC and CD by applying propensity score matching to equalize the groups, with a *p* value < 0.05 indicating a statistical difference.

**Table 3:** Correlation analysis between the worries and age and gender.

**Item**	**Age**	**p** ** value**	**Gender**	**p** ** value**
Worried about going out	0.045	0.304	−0.028	0.513
Worried about travel	0.033	0.448	−0.039	0.371
Worried about taking the bus	0.078	0.074	−0.039	0.366
Worried about not finding public washrooms	0.039	0.375	−0.060	0.166

*Note:* The data in the table represented the correlation coefficient and *p* value.

**Table 4:** Comparison of worries among IBD patients about not finding public washrooms.

	**IBD (cases, %)**	**UC (cases, %)**	**CD (cases, %)**	**Chi-square**	**p** ** value**
Clinical active disease	452 (85.4%)	221 (90.9%)	231 (80.8%)	10.941	0.001
Clinical remissive disease	247 (46.7%)	130 (53.5%)	117 (40.9%)	8.365	0.004

*Note:* The data in the table represented the number of patients expressing worries about not finding public washrooms and the percentage in the total number of each group (patients with IBD, UC, or CD). Statistical evaluations between UC and CD were conducted using the chi-square test.

## Data Availability

The data used to support the findings of this study were available on request from the corresponding author. The data were not publicly available due to privacy or ethical restrictions.
