# Supplementary material for: The Worries of Out-of-Home Activities in Patients With Inflammatory Bowel Disease: A Survey Study During the COVID-19 Pandemic
Source: Gastroenterol Res Pract. 2024 Jul 3;2024:6634377. doi: 10.1155/2024/6634377 (PMC11236464; doi:10.1155/2024/6634377)
Supplement: Supporting Information — Additional supporting information can be found online in the Supporting The English version of the questionnaire. [file 6634377.f1.docx]

**Supplementary Table.**

The English version of the questionnaire.

| **Items** | **Answer** |  | **The Answerer** |  |
| --- | --- | --- | --- | --- |
| Age |  |  | Participants |  |
| Gender |  |  | Participants |  |
| Type of IBD | UC | CD | Chief physician |  |
| Disease activity | Clinical activity (PMS score >= 3); Clinical remission (PMS score of 0 to 2) | Clinical activity (HBI >= 5); Clinical remission (HBI score of 0 to 4) | Chief physician |  |
| Previously diagnosed with anxiety? | Yes | No | Participants |  |
| Previously diagnosed with depression? | Yes | No | Participants |  |
| Scores of worry levels of out-of-home activities  (1=No worried; 2= A bit worried; 3=Moderate worried; 4=Very worried; 5=Most Worried) | | | | |
| Worried about going out |  |  | Participants |  |
| Worried about travel |  |  | Participants |  |
| Worried about taking the bus |  |  | Participants |  |
| Worried about not finding public washrooms |  |  | Participants |  |
